# Doubles Connected Moments Expansion: A Tractable Approximate Horn–Weinstein Approach for Quantum Chemistry

**DOI:** 10.1021/acs.jctc.3c00929

**Published:** 2023-12-05

**Authors:** Brad Ganoe, Martin Head-Gordon

**Affiliations:** Pitzer Center for Theoretical Chemistry, Department of Chemistry, University of California, Berkeley, California 94720, United States

## Abstract

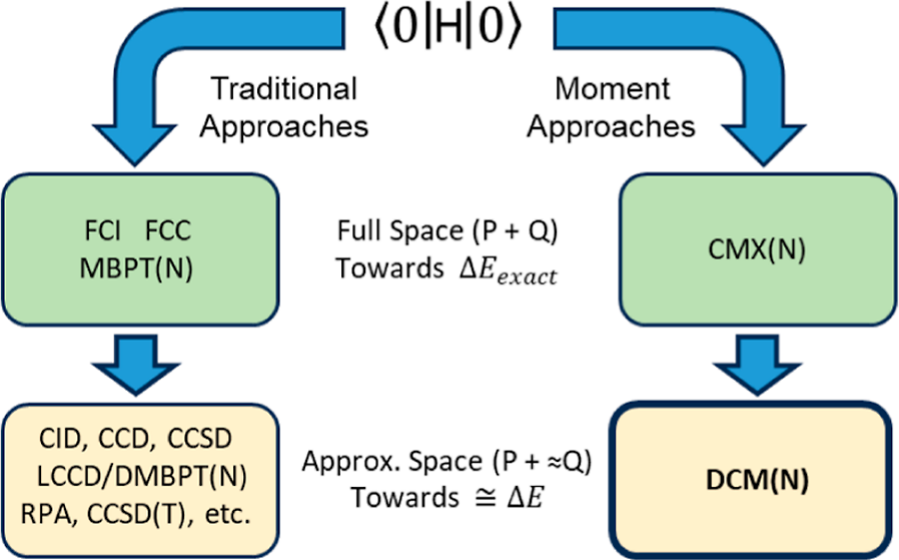

Ab initio methods based on the second-order and higher connected moments, or cumulants, of a reference function have seen limited use in the determination of correlation energies of chemical systems over the years. Moment-based methods have remained unattractive relative to more ubiquitous methods, such as perturbation theory and coupled cluster theory, due in part to the intractable cost of assembling moments of high-order and poor performance of low-order expansions. Many of the traditional quantum chemical methodologies can be recast as a selective summation of perturbative contributions to their energy; using this familiar structure as a guide in selecting terms, we develop a scheme to approximate connected moments limited to double excitations. The tractable Doubles Connected Moments [DCM(*N*)] approximation is developed and tested against a multitude of common single-reference methods to determine its efficacy in the determination of the correlation energy of model systems and small molecules. The DCM(*N*) sequence of energies exhibits smooth convergence toward limiting values in the range of *N* = 11–14, with compute costs that scale as a noniterative *O*(*M*^6^) with molecule size, *M*. Numerical tests on correlation energy recovery for 55 small molecules comprising the G1 test set in the cc-pVDZ basis show that DCM(*N*) strongly outperforms MP2 and even CCD with a Hartree–Fock reference. When using an approximate Brueckner reference from orbital-optimized (oo) MP2, the resulting oo:DCM(*N*) energies converge to values more accurate than CCSD for 49 of 55 molecules. The qualitative success of the method in regions where strong correlation effects begin to dominate, even while maintaining spin purity, suggests this may be a good starting point in the development of methodologies for the description of strongly correlated or spin-contaminated systems while maintaining a tractable single-reference formalism.

## 1 Introduction

Most quantum chemical methods begin from the Schrödinger equation, *H*|Ψ⟩ = *E*|Ψ⟩, and make approximations to find an acceptably accurate expectation value of the Hamiltonian, ⟨*E*⟩ = ⟨Φ|*H*|Φ⟩. As the approximate wave function |Φ⟩ approaches the exact ground-state wave function |Ψ⟩, our approximate expectation value approaches the true energy: ⟨*E*⟩ → *E*. Fine tuning this approach takes many forms but generally begins from a mean-field reference wave function, |Φ_0_⟩, such as the Hartree–Fock (HF) determinant or an approximate Brueckner determinant, with energy *E*_0_ = ⟨Φ_0_|*H*|Φ_0_⟩. One then seeks to approximate the correlation energy, *E* – *E*_0_, of the system to approach the exact energy or values that are within the “chemical accuracy” for various systems.

Perturbation theory (PT) approaches are widely used, in which the correlation energy of the system is expressed as an infinite series^1^
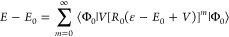
1where *H* = *F* + *V*, the resolvent is , and the choice of parameter ε ≠ *E*_*j*_ defines the type of perturbation theory.^1,2^ Choosing ε = *E*_0_ defines the widely used Rayleigh–Schrödinger^3^ PT, which becomes Møller–Plesset (MP) PT^4,5^ when performed with a HF reference. Alternatively, choosing ε = *E* defines Brillouin–Wigner (BW)^6,7^ PT, which is not size-consistent when performed with a HF reference (see ref (8) for an exception).

The coupled cluster (CC) expansion^9−12^ is obtained through the use of an exponential wave function ansatz, |Ψ⟩ = *e^T^*|Φ_0_⟩, and its projection onto the reference and a set of substituted or excited determinants. One may also explore the Hilbert space variationally such as in the CI, selected CI,^13^ CASSCF,^14^ and DMRG^15^ schemes. While approaching the exact ground-state energy is the primary target of all these approaches, other expectation values can arise, such as ⟨*H*^2^⟩ = ⟨Φ|*H*^2^|Φ⟩ in the variance, or second moment, of the Hamiltonian
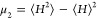
2In the variational methods, μ_2_ → 0 as ⟨*E*⟩ → *E* (and as |Φ⟩ → |Ψ⟩). The variance also plays a role in the CC equations^16^ as follows from inserting the identity (1 = *P* + *Q*, where *P* = |Φ_0_⟩⟨Φ_0_|) in eq 2 to obtain
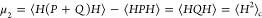
3The CC amplitude equations are solved using *QH̅P* = 0, where  is the similarity-transformed Hamiltonian. As a result, μ_2_ = 0 within the space in which the cluster equations are solved.

Moments play a central role in an alternate approach, the so-called *t*-expansion of Horn and Weinstein.^17^ This approach begins from the observation that a given approximate ground-state wave function, |Φ⟩ ≡ |Φ(*t* = 0)⟩, can always be improved by defining
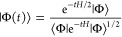
4
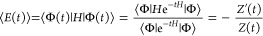
5where *Z*(*t*) is a Maclaurin series containing expectation values of powers of the Hamiltonian operator, ⟨*H*^*j*^⟩ = ⟨Φ|*H*^*j*^|Φ⟩
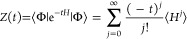
6*Z*′(*t*) is defined as
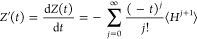
7

The expression for ⟨*E*(*t*)⟩ above is the negative of the logarithmic derivative of *Z*(*t*), and, like propagating in imaginary time, is exact as *t* → ∞. Corresponding expressions ⟨*O*(*t*)⟩ can be obtained for other operators, *O*, that are likewise exact as *t* → ∞.^17^

The promise of the *t*-expansion is that for an arbitrary reference |Φ⟩ satisfying ⟨Φ|Ψ⟩ ≠ 0, the exact energy can be approached not only by the conventional variational approach of improving the trial function |Φ⟩ but also can be attained by deriving an energy expression in terms of coefficients μ_*j*_ that involve powers of the Hamiltonian, evaluated with a fixed reference wave function (like |Φ_0_⟩)
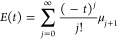
8

Simple powers of the Hamiltonian, ⟨*H*^*j*^⟩, scale as powers of the system volume, *V*^*j*^, while of course the energy is linear in the volume (extensive). Thus, the coefficients μ_*j*_ that enter the expansion above, defined by the negative quotient of the two power series, *Z*′(*t*) and *Z*(*t*), must also scale linearly in the volume. These μ_*j*_ coefficients are the connected moments, , which may be defined in the diagrammatic sense of connectedness or equivalently by evaluating the power series quotient to obtain them one by one. Thus, μ_1_ = ⟨*H*⟩, μ_2_ = ⟨*H*^2^⟩ – ⟨*H*⟩^2^, , etc., and in general
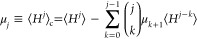
9

Turning the *t*-expansion into a practical computational method is a nontrivial challenge. Creating regular expressions for this series has resulted in various extrapolations.^18,19^ For instance, in the quantum chemistry context, connected moments through μ_12_ were used in Padé approximants to approach the *t* → ∞ energy of the H_8_ model system in the STO-3G basis.^20−22^ At lower order, among the most promising approximations is the connected moments expansion (CMX).^23,24^ A useful form of the CMX can be obtained by presuming that an *N*^th^ order approximation to *E*(*t*), eq 8, can be expressed as a sum of (*N* – 1) decaying exponentials, , and obtaining the coefficients by matching low-order terms in the power series expansions.^24^ In the resulting CMX models, an *N*^th^ order approximation, CMX(*N*) to the ground-state energy may be succinctly written^24^ in terms of the lowest 2*N* – 1 connected moments and the inverse of an (*N* – 1) × (*N* – 1) matrix constructed from those moments
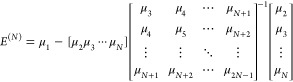
10

In particular, CMX(1) is simply the energy of the reference (i.e., μ_1_), and the two lowest order corrections to the reference energy, CMX(2) and CMX(3), have energies, respectively, defined as
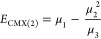
11and
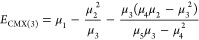
12CMX(4) requires moments up to μ_7_ and the inverse of a 3 × 3 moment matrix. While guaranteed size extensive, creating sums over decaying exponentials removes strict separability. Size consistency is not preserved order-by-order, as in the MBPT case, as these contributions now exist within higher-order terms. Several variants^25,26^ have also been developed to help get rid of the original expansion of singularities and combine the terms in different combinations.

The *t*-expansion is not the only place these connected moments have been the central quantity in determining the energy. Löwdin’s implicit energy formula^27^
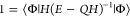
13may be expanded^28^ using the identity
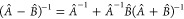
14to obtain an approximate expression for the roots of the energy
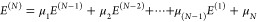
15This set of equations requires the *N* lowest connected moments to evaluate the *N*^th^-order approximation to the energy in the denominator-free perturbation theory.^28^ At second order, the result is . If one optimizes the constant energy denominator associated with the first-order wave function, the CMX(2) result is recovered at second order, with the third-order correction going to zero.

The CMX-based moments approaches and alternatives have been investigated across both model^19,29−39^ and real^26,29,31,40−44^ systems in lattice gauge theory, quantum chromodynamics, and quantum chemistry, which has been well reviewed by Amore.^45^ The convergence and analytic behaviors of these methods have been the focus of several studies in the literature and their strengths and limitations have been well evaluated.^25,31,40,46−48^ Focusing on the problem of the correlation energy of molecular systems, moment-based methods have remained a niche topic of research relative to standard CC or many-body approaches based on the Schrödinger equation, with only a small catalogue of papers using moments-based methods for electronic structure.^20,24,26,28,33,49,50^ Of particular note is the method of moments coupled cluster (MMCC) approach,^51,52^ which acknowledges that the exact energy may be retrieved from any CC reference with energy *E*_CC_ via the formalism of β-nested equations by projecting onto the asymmetric energy expression
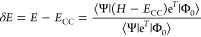
16This yields approximate noniterative corrections through better approximations to |Ψ⟩ by including terms related to the excitation space neglected in the CC amplitudes but extant in the general moment contributions to the exact energy.^53^ For example, use of a CCSD wave function in MMCC requires only up to hextuply excited configurations; while the triple excitations and higher do not factor into the amplitude equations and/or second moment, they do survive in the exact representation of eq 16.

One reason for the dearth of investigation is the cost of assembling these connected moments and the order in which one needs them to approach quantitative accuracy. Early correlation energy studies^24,54^ compared the lowest order CMX expansions to the similar Møller–Plesset series. In weakly correlated systems, the CMX(2) underperforms the MP2 energy while requiring greater computational costs. Yet, assembling μ_3_ scales  with molecule size, *M* (and fixed choice of AO basis), which is the most costly step in the evaluation of CMX(2) with the HF reference. In fact, CMX(2) is fully equivalent to the Unsöld approximation to MP2,^28^ which helps explain this underperformance in the weakly correlated region (although the Unsöld approximation does not diverge for stretched bonds using restricted orbitals, unlike MP2). Going to the next order, CMX(3) depends on μ_5_, whose assembly scales  (the same scaling as full CCSDT). We also note that there has been recent interest in the connected moments as an approach for performing molecular electronic structure calculations on quantum computers.^55−58^

Efforts to truncate the exact full CI (FCI) problem in a computationally tractable way have led to many different approximations that blend elements of CC and PT approaches. The random phase approximation (RPA) is equivalent to an infinite sum over the ring diagrams in the perturbation expansion.^59^ The MP series summed over all possible doubly excited states yields DMBPT(∞),^60^ which is formally equivalent to CEPA(0)^61^ and linearized CCD.^11^ Perturbative analysis^62^ of CCD and CCSD, etc.^63,64^ has guided construction of perturbative corrections to CC energies, with a notable example being CCSD(T)^65,66^ (as well as other alternatives^67,68^ including MMCC which was already discussed^51,52^). Several approximations reliant on removing terms in the series have arisen and have been well reviewed recently.^69^

Despite the success of these selective summations in perturbation theory, no comparable scheme has been reported in approximating connected moments for use in CMX-based methodologies. Keeping in mind what has been successful in the perturbative case, we report a methodology to approximate the connected moments using a selective summation constrained to the doubly excited manifold and apply these approximate connected moments in place of their vastly more expensive exact counterparts. We design a recursive algorithm to construct doubles connected moments and assess the resulting Doubles Connected Moments [DCM(*N*)] energies for approximating correlation energies across a range of small molecules. We report stable results up through 20th order in the DCM(*N*) expansion.

## 2 Doubles Connected Moments Approximation

To begin evaluating finite-order CMX approximations, we must specify a reference from which to construct the connected moments. In this work, we shall take as references both the Hartree–Fock determinant, |Φ_0_⟩ = |Φ_HF_⟩, and the single determinant generated via orbital optimized (OO) MP2,^70−74^ |Φ_0_⟩ = |Φ_OOMP2_⟩. The latter is a simple approximation to the exact Brueckner determinant which is the best single reference.^75,76^ Unlike the case of CCSD, in which we expect orbital insensitivity due to the Thouless theorem,^18^ DCM(*N*) does not have the orbital relaxation effects associated with singles, so meaningful energy changes may be expected when we change reference. DCM(*N*) also benefits from the fact that the (exact) Brueckner determinant maximizes overlap between the reference and exact wave functions. The convergence of the CMX series relies on that overlap and a closer starting determinant should therefore more rapidly approach the exact energy.^36^

We can then define  (note that all connected moments above μ_1_ are invariant to adding a constant to ) using the expectation value of the determinant, *E*_0_, as the reference result in the CMX(1) energy. It may be beneficial to discuss the nature of the low-order canonical case qualitatively before developing the general semicanonical all-order expressions. Going further in the series, one can construct^54^
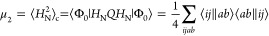
17as the second-order connected moment for the canonical orbitals, with ⟨*ij*∥*ab*⟩ being the antisymmetrized two-electron matrix elements in standard notation.^77^ In particular, double substitutions from occupied orbitals *i*, *j*, *k*, ... to virtual orbitals *a*, *b*, *c*, ... are the only part of *Q* that makes nonzero contributions to μ_2_ in Hartree–Fock. The next moment, μ_3_, also involves only contributions from double substitutions in the cannonical case leading to^54^
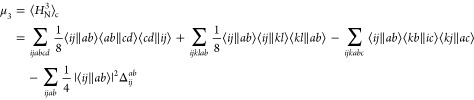
18where Δ_*ij*_^*ab*^ = ϵ_*i*_ + ϵ_*j*_ – ϵ_*a*_ – ϵ_*b*_ are the orbital eigenenergy differences. These one-particle terms now lie in the numerator of only the final term, in contrast to their position in the denominator for all terms in perturbation theory. Having through μ_3_, this makes canonical CMX(2) an exclusively doubles theory. Higher-order moments may be evaluated via the same scheme, with increasingly large subspaces of *Q* making contributions. Specifically, while *H*_N_|Φ_0_⟩ contains up to double substitutions, *H*_N_*H*_N_|Φ_0_⟩ contains single, double, triple, and quadruple substitutions (S,D,T,Q). Therefore, like the MP4 energy, μ_4_ also contains S,D,T,Q contributions and requires  compute effort. μ_4_ also contains one “MP2-like” term with two Fock operators and three “MP3-like” terms with a single Fock operator after combining the complex-conjugate cases.

Similar to MP5, μ_5_ requires  computational effort to evaluate nine classes of terms (SS, SD, ST, DD, DT, DQ, TT, TQ, QQ), plus the set of Δ-containing terms, to enable construction of the CMX(3) energy via eq 12. Higher terms require yet greater computational effort, analogous to the corresponding Møller–Plesset energy terms^5,62^ but without orbital energy difference denominators. With the system-scaling increasing by one power per order of CMX, and the number of distinct terms or diagrams exploding, it is clear that while direct implementation of CMX(3) is challenging, CMX(4) which requires μ_7_, is essentially prohibitive.

There is resemblance between the connected moments, , and the corresponding *j*th order Møller–Plesset energy, , involving the same power of the fluctuation potential yet also key differences. The most obvious distinction is the inclusion of Fock terms in *H*_N_ and the lack of the resolvent *R*_0_ operator for μ_*j*_, which instead features a projection operator, *Q*, in the orthogonal space. These eliminate Møller–Plesset energy eigenvalue denominators in exchange for inclusion in some numerators as shown in eq 18. Instead of (*j* – 1) contributions involving Δ^–1^ in all terms of the *j*th order energy, there are terms containing up to Δ^*j*–2^ in the *j*th connected moment. We note the advantage of additive separability of the Δ-containing moment numerator terms compared with the perturbation theory energy denominators. The use of *Q* over *R*_0_ also requires no determination of a small parameter and may help avoid perturbation theory breakdowns as observed in prior studies of low-order CMX(*N*).

Diagonal Fock components are typically the dominant value in the evaluation of ⟨Φ_*ij*_^*ab*^|*H*_N_|Φ_*kl*_^*cd*^⟩. This fact is also why MP2 recovers most of the correlation energy in weakly correlated (large orbital gap) systems. In such systems, because the gap is large, so too is  and therefore the doubles amplitudes are small relative to one: . Letting the largest double amplitude have magnitude *t*_max_ ≪ 1, we then expect the magnitude of the MP3 energy to be smaller than MP2 by roughly this same factor because it involves one more power of *R*_0_*V*. For MP4, the connected triples and quadruples give rise to larger eigenenergy differences such as Δ_*ijk*_^*abc*^ and Δ_*ijkl*_^*abcd*^ which is compounded with the presence of one more power of *R*_0_*V* in the MP4 energy. Connected doubles tend to dominate in single-reference cases, which is one of the strengths of the CC methods, as its most basic form functions as a resummation of these terms through infinite order. Heuristically, the energetic contributions of the singles and triples cancel against the quadruples, contributing another advantage that has led to the success of even low-order perturbation theory and methods constrained to only doubles.

We now introduce approximations in the construction of various higher-order moments by exploiting these very same arguments, likewise considering the single-determinant case. In this instance, the dominant term in μ_3_ ought to be the MP2-like term mentioned above. In μ_4_, the MP2-like term scaling as  is roughly an order of magnitude larger than the MP3-like terms scaling as Δ_*ij*_^*ab*^, and so forth. Though we lack the benefit of inverse energy difference decay, we see that at each *N*-th order cumulant, the largest expected term is always contained in the connected doubles, keeping the comparison to the PT relevant. Going outside the single reference case, these terms may cease to be the largest. However, unlike in Møller–Plesset where near degeneracies cause a singularity in the equations, the terms do not diverge in the connected moments and merely tend toward zero. This leaves the dominant small-gap terms as those containing no Fock operators. This suggests that evaluating the connected moments within just the subspace of doubles would be a worthwhile venture just as it has been in the case of CC and PT theory.

In the case of semicanonical orbitals, the exact μ_2_ includes an additional term corresponding to a sum over the Fock contributions from non-Brillouin singles, , yet ameliorated in magnitude by their approximate Brueckner-like nature. Non-Hartree–Fock reference determinants would have these single contributions neglected in such an approximation, which otherwise would only begin formally at μ_4_. Similar use of these orbitals in work on perturbation theory had found that approximations neglecting these singles contributions at third order are sufficient in the construction of low-order energies.^78,79^ As the CMX matrix equation is of a more complex form than the sum, we desire to keep an even-tempered approach to the description of the Hilbert space at each level of the moments. Thus, we retain only doubles at all orders in this description of both the canonical and semicanonical case of our equations.

The doubles approximation to the connected moments will be defined by replacing the resolution (*P* + *Q*) by (*P* + *D*) where *D* is the doubles subspace of the orthogonal Hilbert space: . In analogy to DMBPT(∞), we can begin by defining the intermediate integral tensors and now incorporating off-diagonal elements of the doubles
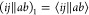
19and
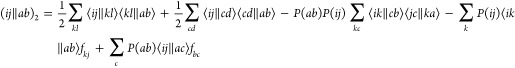
20where *P*(*pq*) is the standard antisymmeterizer function for electrons *p* and *q*, (*ij*∥*ab*)_1_ is identical to a raw antisymmeterized two-electron integral and the terms in (*ij*∥*ab*)_2_ comprise the hole ladder, particle ladder, ring term, and the “MP2-like” delta terms of μ_3_, respectively, which takes the form of −⟨*ij*∥*ab*⟩Δ_*ij*_^*ab*^ in the Hartree–Fock case. From here, we may generate the 20 skeletal diagrams contributing to the μ_4_ doubles: 16 reminiscent of the doubles contribution to MP4, as well as three MP3 like terms with a single Fock contribution and one MP2 like term with two Fock contributions by a simple recursion
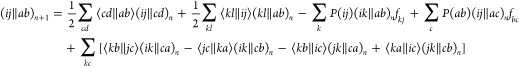
21

All terms of higher order may be generated directly from the previous intermediate. Simple contraction of these intermediates results in the doubles approximation to the connected moments (DCM)
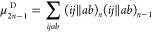
22and
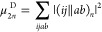
23Like DMBPT(∞) or even CCD and CCSD, the limiting step in the formulation of the intermediates is the  particle ladder contraction, similar to the MP3 energy or the CCD/CCSD amplitudes equation. However, unlike CCD/CCSD, this does not require solving a nonlinear set of equations, and outside of the recursion, no iterations for the determination of amplitudes or energies are needed in the formation of the intermediates. One may then use the values of the approximate moments μ_2*n*–1_^D^ in place of the exact μ_2*n*–1_ to yield the DCM(*N*) approximations to the full CMX(*N*) model.

As MBPT(*N*) and CMX(*N*) both approach the exact energy as *N* → ∞, the relation of DCM(∞) to other double-excitation methods is of interest. For DMBPT(∞), this is exactly a linearized version of the CCD equations. LCCD has also been motivated as a size-extensive correction to CID, whereby inclusion of unlinked terms to the CID energy for proper treatment under the linked-diagram theorem yields exactly the LCCD amplitude equations (notably enforcing μ_2_ = 0) and a lower bound on the CID energy.^80^ No such similar or deeper relations are currently known for DCM(∞) or other approximate moment methods based on the imaginary time propagator. This is an open issue of interest.

## 3 Implementation

The code necessary to form (*ij*∥*ab*)_*n*+1_ and therefore the necessary doubles connected moments, μ_2*n*–1_^D^, μ_2*n*_^D^, was implemented in a development version of the Q-Chem quantum chemistry program,^81,82^ which has also been used to carry out the all-electron calculations for each methodology in the following examples. Two other important aspects of the implementation should be mentioned.

First, unlike in the PT case, higher-order moments do not generally shrink in value, except in the (rather pointless) case of an exact reference function, for which the second order and higher connected moments all evaluate to zero. However, μ_*k*_ has dimensionality *E*^*k*^ and is dominated by terms with , whose largest value is
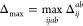
24

We then redefine the energy scale such that
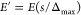
25to keep the moments from exploding in value. *s* is a dimensionless scale factor (*s* ∼ 1) that will be explored and then selected. In turn, the scaled energy can be unscaled after the CMX algorithm is applied to obtain the final DCM(*N*) energy. This procedure ameliorates the round-off error accumulation due to operations on quantities with greatly differing magnitudes.

Second, we do not explicitly invert the (*N* – 1) × (*N* – 1) matrix of moments that enters eq 10. Rather, we solve a set of linear equations
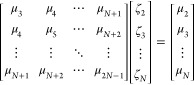
26and then evaluate
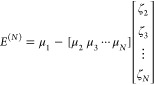
27This improves numerical stability in the case where the coefficient matrix may be near singular.

Having discussed the necessity of scaling, we next present some specific data for water in cc-pVDZ. As a reference point, the unscaled approximate moments from μ_4_ to μ_39_ span a range of over 57 orders of magnitude! For various scaling factors (*s* in eq 25) between 0.85 and 1.25, we show the logarithm of the scaled range of the DCM(*N*) moments and the energy differences (in μH) between calculated DCM(*N*) energies in Table 1. The energy differences are defined as Δ(*N*, *s*) = [DCM(*N*)](*s*) – [DCM(*N*)](1.1) and illustrate how numerically stable the calculations are, since in infinite precision all Δ(*N*, *s*) values are zero.

**Table 1:** Differences (in μH), Δ(*N*, *s*) = [DCM(*N*)](*s*) – [DCM(*N*)](1.1) (*N* = 14...20), between DCM(*N*) Energies for Water Evaluated with Various Scaling Factors, *s* (Defined in Eq 25), Relative to Values Evaluated with *s* = 1.1^a^^b^

	scaling factor (*s*)							
difference	0.85	0.90	0.95	1.00	1.05	1.15	1.20	1.25
Δ(14, *s*)	119.5	127.9	–6.0	–6.0	–4.4	–0.4	0.8	1.0
Δ(15, *s*)	138.6	–12.5	–8.8	–6.8	–3.6	2.7	5.7	10.1
Δ(16, *s*)	–25.2	–20.5	–15.6	–10.7	–5.5	5.6	12.0	18.1
Δ(17, *s*)	35.0	42.2	49.9	58.1	–3.2	1.7	4.9	9.0
Δ(18, *s*)	38.2	49.1	–11.8	–8.3	–4.6	2.7	7.3	10.5
Δ(19, *s*)	47.1	63.4	–13.2	–9.3	–5.0	4.5	9.0	13.8
Δ(20, *s*)	59.8	–20.6	–15.4	–10.7	–5.3	5.6	11.1	15.5
log (range)	3.66	2.80	1.97	1.19	0.52	1.18	1.78	2.36

a The logarithm of the range of calculated double moments in scaled energy units is also shown for each choice of s.

b Logarithmic range of 1.1 is 0.57.

The range of magnitudes of the scaled connected moments is vastly improved (<10^4^) for all scalings (*s*) considered in Table 1. Even using the unscaled energies, all energies through DCM(5) agree with values obtained using all choices of scaled units to within machine precision. Showing the value of using eq 25, agreement to virtually machine precision is still obtained for all choices of *s* tested here through DCM(11) while direct use of atomic units becomes unstable. However, for *N* ≥ 14, these higher-order DCM(*N*) energies show differences of  and suggests that solving the linear equations, eq 26, may involve some ill-conditioning in double precision, even in scaled energy units.

To assess whether or not eq 26 can be ill-conditioned, Table 2 shows the logarithm of the condition numbers for both the H_2_O and F_2_ systems. Quite plainly for higher orders of DCM(*N*) or oo:DCM(*N*), the condition number becomes large enough that the maximum forward–backward error can, in theory, make the results meaningless in double precision (64 bit) arithmetic. For instance by DCM(13), the condition number is larger than the inverse of 64 bit machine precision (∼10^15^). Despite this, the error in-practice is the quantity of interest, and many linear algebra techniques are widely employed in the context of poorly conditioned matrices. Table 3 contains errors of DCM(*N*) from the exact FCI for the fluorine dimer for various linear algebra approaches using moments evaluated with double precision. The only difference between trials is small perturbations at the edge of double precision. As will be examined in more detail later, fluorine has the largest fluctuations between DCM(*N*) orders we have observed. As is evident from Table 3, the DCM(*N*) errors exhibit a sawtooth pattern with trough-to-peaks of about 0.8 mH when utilizing Armadillo with a Moore–Penrose pseudo-inverse (xGELSD) algorithm,^83^ a common fallback strategy for poorly conditioned matrices. This may be compared to other approaches, such as the standard xGESV class of subroutines in LAPACK^84^ or the NumPy^85^ routines, likewise based on the xGEEV subroutines.

**Table 2:** Logarithm of the Condition Number of the Coefficient Matrix in Equation 26, for DCM(*N*) and oo:DCM(*N*) (*N* = 14...20) for H_2_O and F_2_

	H_2_O		F_2_	
order (*N*)	HF	OO	HF	OO
DCM(6)	5.66	5.66	5.88	5.89
DCM(9)	10.28	10.28	10.41	10.40
DCM(10)	12.34	12.34	12.39	12.39
DCM(11)	13.56	13.55	13.53	13.52
DCM(12)	15.07	15.18	14.98	14.96
DCM(13)	15.65	16.39	15.56	15.51
DCM(14)	16.01	16.78	15.45	15.74
DCM(15)	15.96	16.82	15.47	15.77
DCM(16)	15.86	16.14	16.33	15.64
DCM(17)	17.67	16.21	16.04	15.68
DCM(18)	15.86	16.14	16.33	15.64
DCM(19)	16.29	16.47	16.16	15.49
DCM(20)	16.13	16.73	16.69	15.89

**Table 3:** Error from the Exact Energy (mH) for F_2_ for Various DCM(*N*) Methods Using 3 Different Linear Algebra Packages to Solve Equation 26^a^

	method					
trial	DCM(11)	DCM(13)	DCM(15)	DCM(17)	DCM(19)	DCM(20)
Moore–Penrose Pseudo-Inverse (xGELSD)						
1	5.608	4.844	5.625	5.028	5.568	5.747
2	5.613	4.873	5.623	5.036	5.568	5.746
3	5.611	4.84	5.622	5.026	5.567	5.747
4	5.601	4.845	5.623	5.026	5.568	5.748
Standard LAPACK (xGESV)						
1	5.608	4.839	4.762	4.795	3.698	3.156
2	5.614	4.863	5.036	4.760	3.319	3.254
3	5.611	4.835	4.911	4.784	2.92	3.437
4	5.601	4.826	4.794	4.799	3.428	3.810
Standard NumPy (xGEEV)						
1	5.610	4.775	4.704	4.832	4.441	4.375
2	5.614	4.862	5.047	4.727	4.402	4.272
3	5.613	4.755	4.844	4.787	4.22	4.477
4	5.600	4.910	4.827	4.85	3.574	4.367

a Trial 1 corresponds to use of moments as calculated in double precision arithmetic while trials 2–4 involve a random perturbation added to the calculated moments at the level of 10^–13^.

With both of the standard algorithms not incorporating techniques to manage singular values, we see a stronger inconsistency between trials. While in all cases the low-order DCM(11) errors are near our 10 μH criteria, by DCM(13) the nonadapted xGESV and xGEEV codes show larger maximum differences between runs of up to 0.78 and 0.87 mH for DCM(19). By contrast, the Moore–Penrose xGELSD algorithm ameliorates this difference to 1 μH at DCM(19), while its largest difference between runs is for DCM(13) at 33 μH.

Based on the data presented, as well as tests on other members of the G1 data set, we decided to employ a scaling factor of *s* = 1.1 in eq 25 to evaluate the moments, followed by multiplying its reciprocal value back to the DCM(*N*) energy in order to rescale back to atomic units. We use the Moore–Penrose SVD through the Armadillo C++ library utilizing xGELSD. These exploratory tests clearly show that there are intrinsic precision challenges associated with the DCM(*N*) approach such that one cannot reliably determine an exact *N* → ∞ limit in double precision: we limit ourselves to a maximum value of *N* = 20.

## 4 Results and Discussion

### 4.1 Size Extensivity and Size Consistency

Since the moments that enter the DCM(*N*) energy expressions are connected, we expect DCM(*N*) to be extensive in the sense that calculations on *m* noninteracting replicas of a molecule, A, lead to *E*(*m*A) = *mE*(A). By contrast, size consistency (*E*(A···B) = *E*(A) + *E*(B), where A and B are infinitely separated in A···B) is not obvious. We therefore performed numerical tests of both properties, and the deviations from extensivity are assessed in Table 4 for well-separated identical noble gas atoms (Ne_2_) while size consistency is assessed for a well-separated Ne–Ar dimer. As expected, the extensivity errors are very small at all orders (not all are shown). By contrast, the size-consistency error is large at the lowest order CMX(2), at 13.47 mH. This jumps significantly in low-order DCM(*N*), with DCM(5) resting 43.86 mH above, before decaying to a 2.06–2.63 mH error in DCM(13)–DCM(20). It appears that high-order terms in DCM(*N*) ameliorate some of the error seen in low order, but complete cancellation does not occur. In summary, neither finite order CMX(*N*) nor DCM(*N*) at any order is exactly size-consistent despite their extensivity. This is a formal and practical disadvantage against standard CC and PT, and we now turn to assessing the performance of DCM(*N*) versus such methods.

**Table 4:** Size-Extensivity Errors (mH) for Ne_2_ and Size-Consistency Errors (mH) for Ne and Ar Atoms for Various DCM(*N*) and oo:DCM(*N*) Orders^a^

	Ne_2_ size-extensivity error		Ne···Ar size-consistency error	
order (*N*)	HF	OO	HF	OO
DCM(2)	2.64 × 10^–^^6^	1.82 × 10^–^^6^	13.47	13.36
DCM(3)	4.18 × 10^–^^6^	1.83 × 10^–^^6^	29.91	29.77
DCM(4)	4.40 × 10^–^^6^	1.83 × 10^–^^6^	26.31	26.19
DCM(5)	4.29 × 10^–^^6^	1.83 × 10^–^^6^	43.86	43.84
DCM(6)	4.30 × 10^–^^6^	1.83 × 10^–^^6^	24.95	24.98
DCM(7)	4.30 × 10^–^^6^	1.81 × 10^–^^6^	8.36	8.39
DCM(8)	3.36 × 10^–^^6^	9.13 × 10^–^^7^	6.49	6.53
DCM(10)	–2.81 × 10^–^^4^	–1.63 × 10^–^^4^	2.54	2.54
DCM(12)	–3.46 × 10^–^^4^	8.13 × 10^–^^5^	2.90	2.91
DCM(14)	–7.45 × 10^–^^5^	–5.51 × 10^–^^5^	2.24	2.23
DCM(16)	3.91 × 10^–^^5^	4.02 × 10^–^^5^	2.63	2.65
DCM(18)	–3.98 × 10^–^^5^	7.19 × 10^–^^6^	2.16	2.20
DCM(20)	6.33 × 10^–^^4^	–5.49 × 10^–^^4^	2.47	2.46

a Each separated system is calculated in cc-pVDZ at 100 Å.

### 4.2 G1 Test Set

We address the accuracy of the DCM method for a wide range of small molecules as a measure of its usefulness for the description of chemical systems. Toward this end, we compare the energies of CMX(2), DCM(*N*), MP2, CCD, CCSD, and CCSD(T) against a very good approximation to full CI. Specifically, we used adaptive sampling configuration interaction (ASCI) with a second-order perturbation correction (i.e., ASCI + PT2)^13,86^ with a cc-pVDZ basis across 55 systems contained in the first-row G1 test set.^87−89^ We note that we use the original geometries of the G1 test set, and we performed the ASCI + PT2 calculations using the implementation in Q-Chem.^82^ All ASCI + PT2 calculations were converged to within 10 μH and serve as an effectively converged full CI calculation. All calculations used the frozen core approximation.

As the nature of the DCM series’ convergence was not known before this work, we will report its behavior for specific systems in addition to the general statistics of the test set. In particular, low-order CMX has been reported to occasionally converge to excited states in the case of a dominant excited state configuration.^90^ Erroneously, CMX was thought to converge to incorrect values in seminal studies,^24^ but it was demonstrated that this was an excited state energy of the system.^45^ In the spectral representation of Horn–Weinstein, as one reaches high orders, all excited states ought to be sufficiently damped away such that only the ground state survives. However, this condition is not necessarily ensured in our subsection of the Hilbert space limited to doubles nor is the monotonic nature of the series.

It will also be a useful task to assess the convergence of the DCM(*N*) series to determine whether the *N*th term improves over the previous value. We next look to inspect some representative systems of DCM(*N*)’s behavior in the G1 test set: H_2_O, F_2_, C_2_H_6_, CN, and Li_2_.

### 4.3 H_2_O and Model Scaling

**Figure 1:**
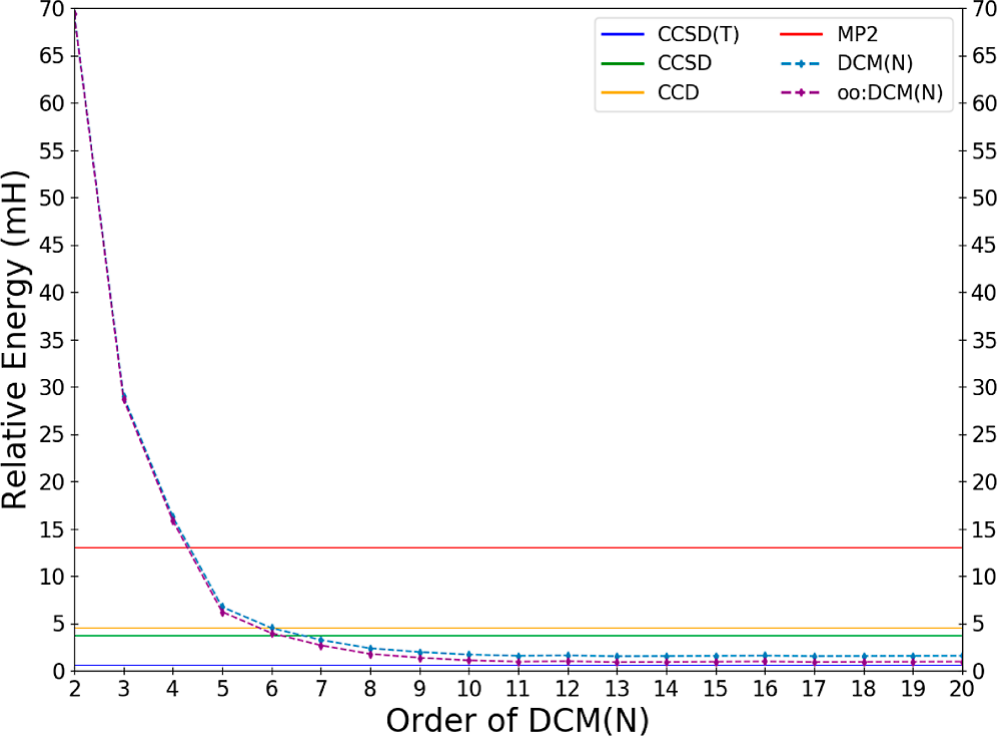
Deviations in the calculated DCM energy of water in the cc-pVDZ basis relative to the effectively exact reference result as a function of the order *N* of the DCM(*N*) and oo:DCM(*N*) sequences.

**Figure 2:**
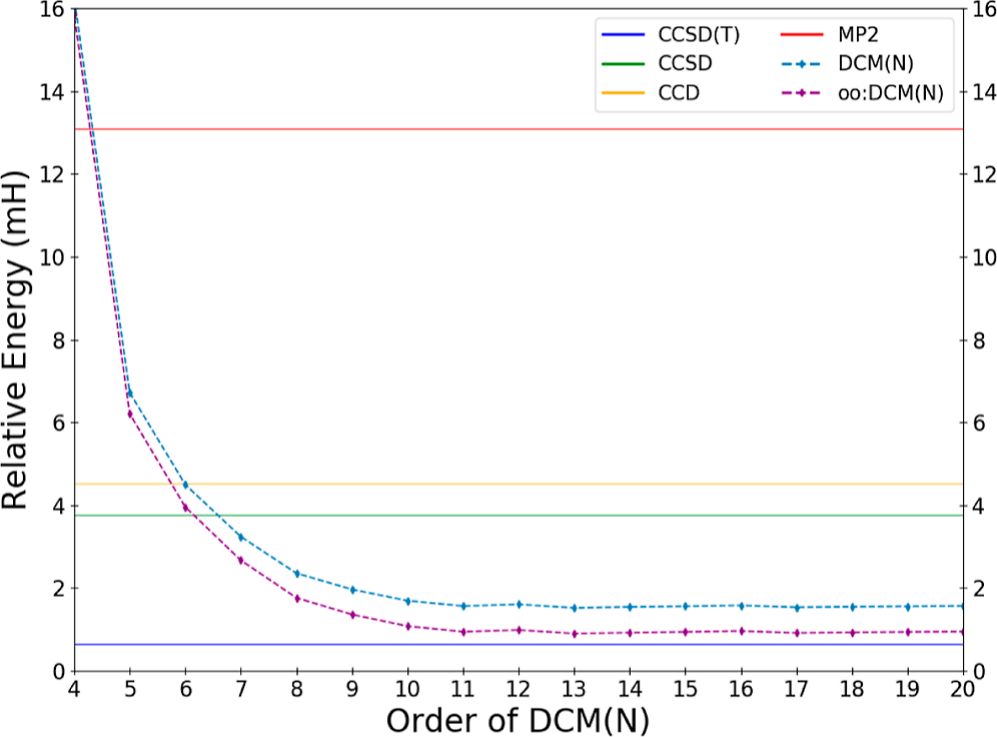
Deviations in the calculated DCM energy of water in the cc-pVDZ basis relative to the effectively exact reference result as a function of the order *N* of the DCM(*N*) and oo:DCM(*N*) sequences for *N* ≥ 4.

Water has served as a test molecule for the behavior of high-order perturbation theory since early algorithms were developed, including sets of selective summations toward the infinite limit which we seek to likewise address.^60^ Therefore, it serves as a useful comparison to begin to understand the behavior of the DCM(*N*) series. In Figure 1, we depict order-by-order the DCM(*N*) and oo:DCM(*N*) energies relative to the exact energy of H_2_O in the cc-pVDZ basis, while a more resolved image focused on just the fourth order and higher terms are depicted in Figure 2. The energy errors of some common methods (MP2, CCD, CCSD, and CCSD(T)) relative to the exact energy are depicted as horizontal lines.

Referring to Figure 1, CMX(2) and oo:DCM(2) are evidently a very poor description of electron correlation, with over a 5 times larger error than the (cheaper) MP2 method. We also see that low-order DCM(*N*) is insufficient for quantitative accuracy; DCM(*N*) is inferior to CCD and CCSD until DCM(6) and DCM(7), respectively. However, the fact that the DCM(*N*) sequence crosses over with CCD and CCSD is exciting and perhaps unexpected. Figure 2 provides a zoomed-in view of the approach of the sequence of DCM(*N*) values toward a potential DCM(∞) limit. The sequence settles down to be close to an apparent limit by DCM(11). There is nonmonotonic behavior with variations in the value differing by less than 0.1 mH until DCM(14); after this all energetic changes are no larger than 30 μH.

This convergence behavior is reminiscent of the best behavior seen in the case of conventional Møller–Plesset perturbative series, such as for water at *R*_e_ in very small basis sets.^91^ However, even well-behaved closed shell systems such as the Ne atom exhibit poor convergence and even divergence in slightly larger basis sets such as aug-cc-pVDZ.^92^ By contrast, the exact CMX series has a monotonic approach to exactness. However, we have no guarantee that our double approximation will replicate the monotonicity of CMX and therefore oscillations are a possibility. In this regard, the data shown in Figures 1 and 2 are very encouraging as the DCM(*N*) sequence appears quite stable, at least up through DCM(20) in the tested cc-pVDZ basis. One can explain this by the fact that the products of small eigenenergies in the denominator contributing to this behavior in perturbation theory go toward zero in the corresponding moments. Most intriguingly, both the HF- and oo- based DCM(*N*) methods outperform their fellow *O*(*N*^6^) CCD and CCSD methods in water. The DCM(*N*) methods even recover a substantial fraction of the triple contribution, particularly when using the OOMP2 reference determinant.

### 4.4 CN and C_2_H_6_

**Figure 3:**
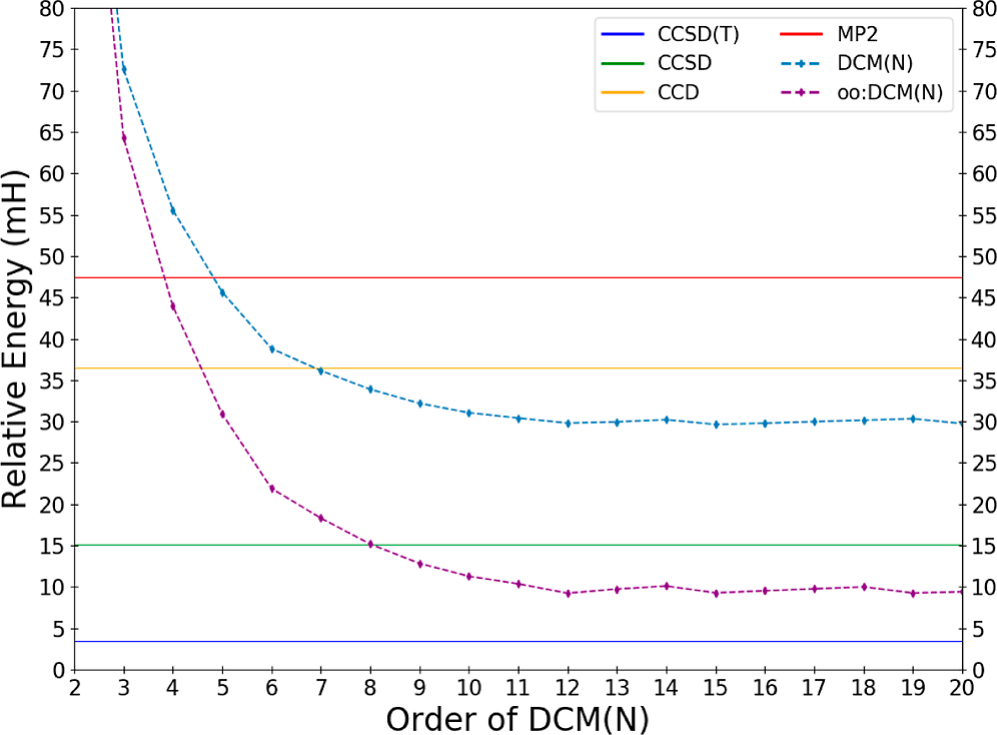
Deviations in the calculated DCM energy of CN in the cc-pVDZ basis relative to the exact full configuration interaction result as a function of the order *N* of the DCM(*N*) and oo:DCM(*N*) sequences.

The cyano radical and ethane present two interesting examples of the behavior of the DCM(*N*) sequence of energies. They are the systems in which DCM(*N*) perform the worst and best compared to CCSD relative to the exact energy. In the case of CN, CCSD outcompetes high-order DCM(*N*) by about 15 mH, with Figure 3 showing the values of DCM(*N*) by order, while for ethane high-order DCM(*N*) outcompetes by about 7.6 mH.

In fact, CN exhibits the largest error across the entire test set for DCM(*N*). We see an immediate clue as to the origin of the error from the fact that CCD shows an even larger error, that is, about 20% worse than the DCM(*N*) sequence and over twice as large as CCSD. If this is truly an issue associated with the lack of singles, approximate Brueckner-like orbitals would help ameliorate the error associated with orbital relaxation. Indeed the much better performance of oo:DCM(*N*) suggests this is the case as the error is reduced by roughly a factor of 3 (or 20 mH), with the correlation energy below that of CCSD by DCM(9). As a result, CN is not the worst performer within the data set for oo:DCM(*N*). This title instead belongs to SO_2_, in which both oo:DCM(13) and oo:DCM(20) have a 31.6 mH error compared to CCSD’s 25.1 mH.

**Figure 4:**
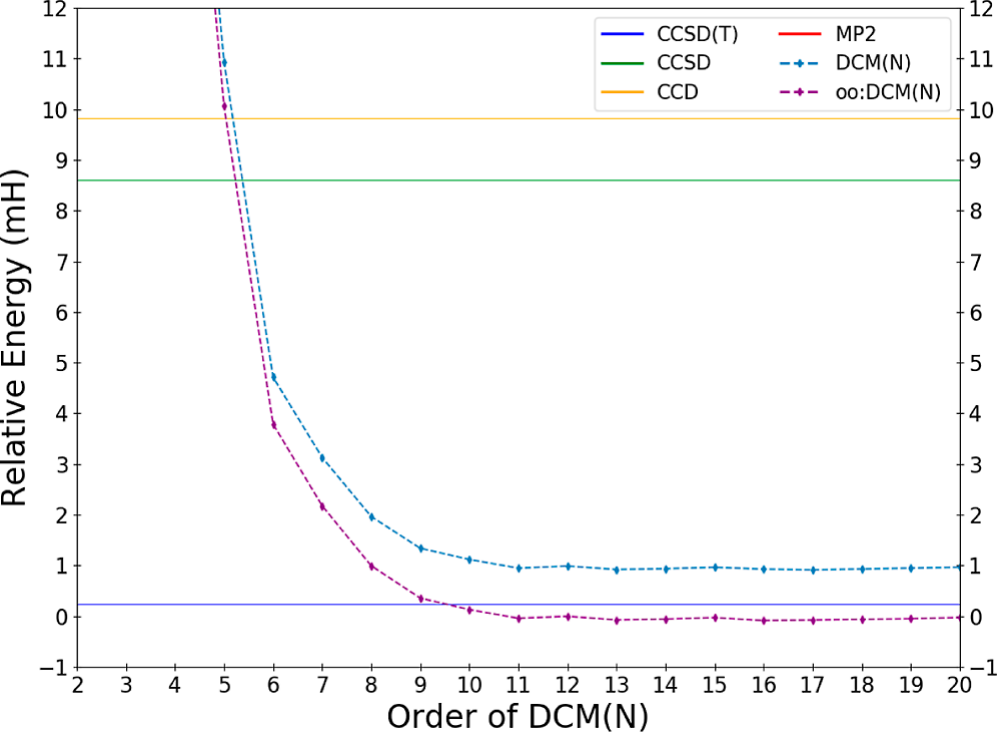
Deviations in the DCM energy of the ethane molecule in the cc-pVDZ basis relative to the effectively exact reference result as a function of the order *N* of the DCM(*N*) and oo:DCM(*N*) sequences.

The ethane molecule presents an interesting contrast in that CCSD is most outperformed by the DCM(*N*) sequence. Figure 4 shows the behavior of the energy error with respect to DCM(*N*) order as before, with CCSD and CCD showing errors of roughly 8 and 9 mH relative to the CCSD(T) level of chemical accuracy. This example is not so much a case of poor performance of either CCD or CCSD but remarkably good performance of the DCM(*N*) sequence. The limiting error of DCM(*N*) is only about 1 mH, and the oo:DCM(*N*) sequence has error that is less than 0.1 mH, and it is clearly superior to CCSD(T), which is in error by 0.24 mH. This example suggests that the importance of triple substitutions in the oo:DCM(*N*) hierarchy may be less than in the usual CC hierarchy.

### 4.5 F_2_ and Li_2_

**Figure 5:**
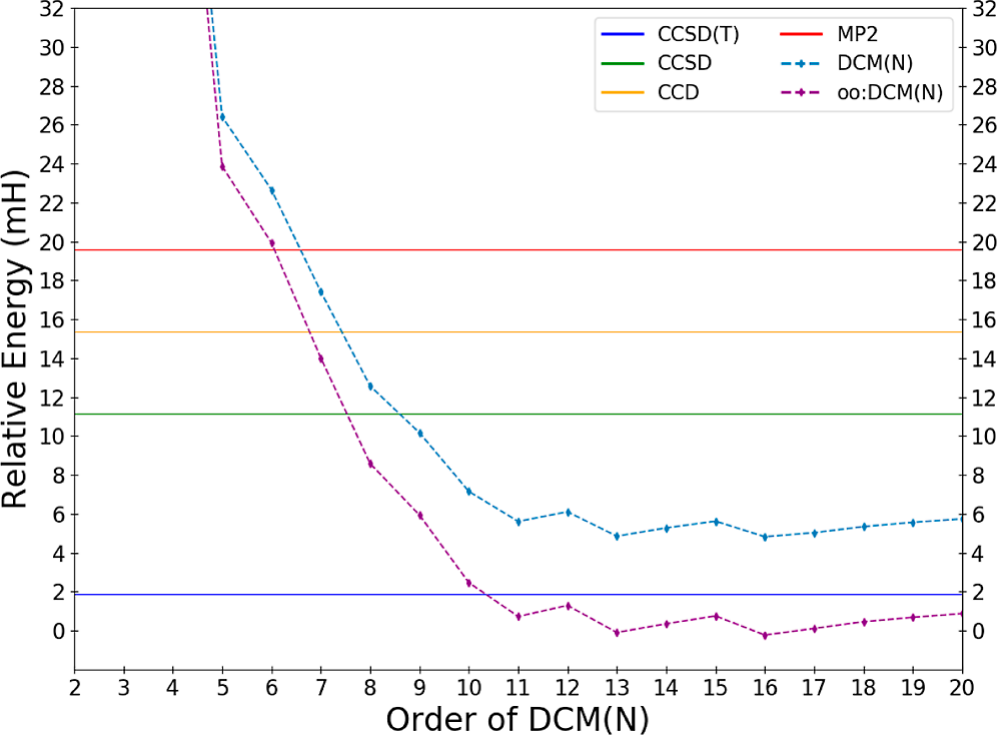
Deviations in the DCM energy of the F_2_ molecule in the cc-pVDZ basis relative to the effectively exact reference result as a function of the order *N* of the DCM(*N*) and oo:DCM(*N*) sequences.

Figure 5 shows the correlation energy errors of the DCM(*N*) and oo:DCM(*N*) sequences relative to several standard methods for the case of the fluorine dimer using restricted orbitals. F_2_ is known to exhibit significant diradicaloid character, and in fact, is not even bound at the mean-field Hartree–Fock level. In this case, we see the most significant oscillatory patterns in the DCM(*N*) energies out of the full set of molecules. Referring back to the investigation of the forward–backward error, we presume that a significant portion of this is due to the choice of SVD algorithm, rather than a true oscillation between the series given infinite precision. This may be elucidated by performing the calculation with increased precision in the moments, as even one or two additional digits would drop the magnitude to within chemical significance for the most severe example we have encountered thus far. Nonsmooth decreases in the correlation energy occur until DCM(11), at which point the more minor changes we saw before swell to span a range of 1.5 mH between the troughs and peaks of oo:DCM(12)/oo:DCM(13) and oo:DCM(16)/oo:DCM(17), with slightly smaller values in the Hartree–Fock reference case. Despite this slightly troubling behavior, the DCM(*N*) values significantly surpass the CCSD energy even before the oscillations occur. In the oo:DCM(*N*) case, the energies even improve upon the costlier CCSD(T) method.

**Figure 6:**
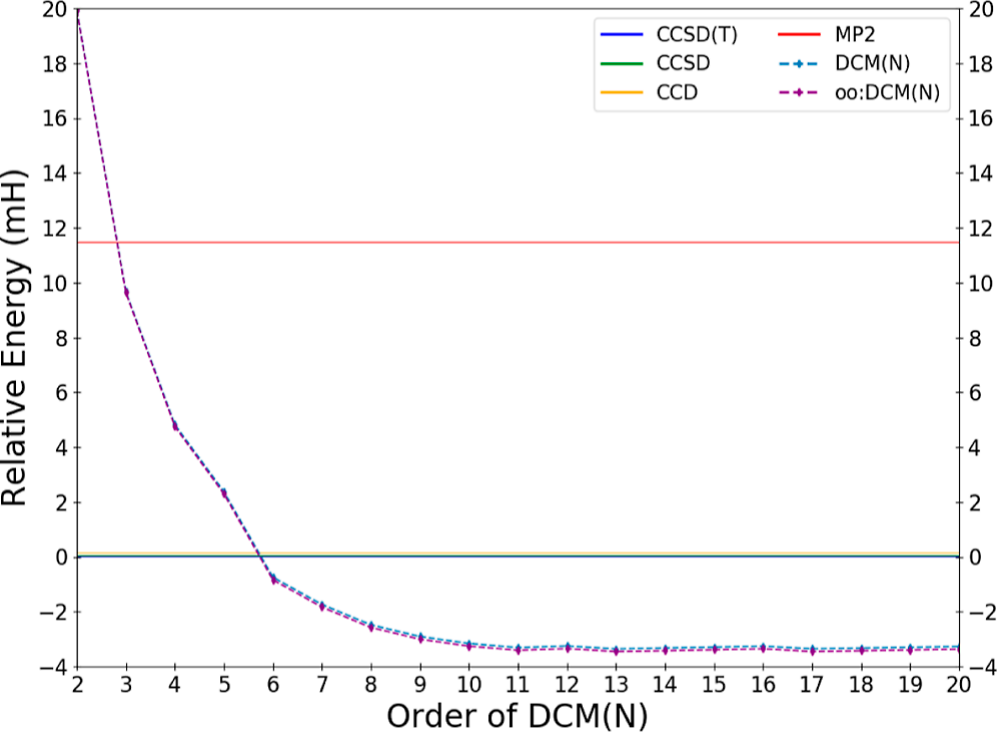
Deviations in the DCM energy of the Li_2_ molecule in the cc-pVDZ basis relative to the effectively exact reference result as a function of the order *N* of the DCM(*N*) and oo:DCM(*N*) sequences.

The lithium dimer exhibits nonvariational behavior for DCM(*N*) energies as shown in Figure 6. While CCD, CCSD, and CCSD(T) are all within chemical accuracy for this effectively two-electron system, both the DCM(*N*) and oo:DCM(*N*) sequences show more than 10 times larger error, with energies going below the exact energy by up to 3.4 mH. The slightly worse behavior in the case of oo:DCM(*N*) suggests that this is not a deficiency associated with the singles. Whether the resummation structure or the moment structure is the cause of this undesirable behavior is unclear to us at present. Nonvariational behavior is observed in LiH also, although the effect is less severe. We note our presentation of the order-by-order analysis for various molecules illustrates that different behavior can arise from one system to the next. We additionally note that it seems to be generally true that the first nonmonotonic values occur between DCM(11) and DCM(14), which is also the range where values appear to also approach limiting values in well-behaved cases.

### 4.6 Results across the G1 Test Set

The statistical results across the G1 test set are contained within Table 5, where the RMSD of the various methodologies discussed are shown for both the Hartree–Fock and OO reference determinants. The data have been arranged with respect to the entire set as well as two subsets: one containing molecules with only row 1 and 2 atoms, followed by the complement subset containing row 3 atoms. As expected, low-order DCM(*N*) energies are not useful methods, in line with our understanding of the poor energetic performance of CMX from past investigations. We also note that there is no improvement, and in fact even slightly poorer performance of oo:DCM(2) and oo:DCM(3) compared to their Hartree–Fock counterparts.

**Table 5:** rmsd of Approximate Methods for the Correlation Energy Relative to (Nearly) Exact Selected Configuration Interaction Results (in mH) across the G1 Test Set, Evaluated in the Cc-pVDZ Basis

	HF rmsd				OO rmsd		
method	all	row 1 + 2	row 3	method	all	row 1 + 2	row 3
CCSD(T)	1.42	1.32	1.53				
CCSD	9.98	9.49	10.58				
CCD	13.60	13.58	13.62				
MP2	27.80	24.93	31.12				
CMX(2)	114.29	101.78	128.67	oo:DCM(2)	115.29	101.72	130.76
DCM(3)	76.69	48.14	102.39	oo:DCM(3)	77.04	46.79	103.79
DCM(4)	55.60	30.96	76.45	oo:DCM(4)	55.34	28.93	77.05
DCM(5)	30.22	18.89	40.40	oo:DCM(5)	29.32	16.07	40.45
DCM(6)	23.44	14.56	31.40	oo:DCM(6)	22.20	11.36	31.02
DCM(7)	19.88	12.26	26.67	oo:DCM(7)	18.50	8.76	26.18
DCM(8)	14.90	10.60	19.07	oo:DCM(8)	13.07	6.86	18.18
DCM(9)	12.46	9.59	15.40	oo:DCM(9)	10.43	5.73	14.38
DCM(10)	11.26	8.90	13.73	oo:DCM(10)	9.16	4.96	12.67
DCM(11)	10.45	8.51	12.53	oo:DCM(11)	8.22	4.54	11.33
DCM(12)	10.31	8.44	12.32	oo:DCM(12)	8.10	4.50	11.15
DCM(13)	10.43	8.32	12.64	oo:DCM(13)	8.27	4.35	11.50
DCM(14)	10.27	8.44	12.23	oo:DCM(14)	8.06	4.47	11.09
DCM(15)	10.33	8.38	12.41	oo:DCM(15)	8.11	4.39	11.22
DCM(16)	10.36	8.24	12.58	oo:DCM(16)	8.19	4.27	11.40
DCM(17)	10.41	8.32	12.61	oo:DCM(17)	8.22	4.35	11.41
DCM(18)	10.37	8.40	12.45	oo:DCM(18)	8.14	4.43	11.24
DCM(19)	10.38	8.43	12.44	oo:DCM(19)	8.15	4.39	11.28
DCM(20)	10.34	8.27	12.51	oo:DCM(20)	8.15	4.28	11.34

Consistent with our examination of individual cases, the most exciting results concern the convergence of DCM(*N*) energies, and especially the oo:DCM(*N*) energies to very useful values by DCM(11–14) or oo:DCM(11–14). Seeking a direct comparison of the purely doubles methods with similar orbitals, we see a notable improvement over the CCD energies as one iterates through the DCM(*N*) cycles across both the whole set and the partitions. This improvement is most significant for row 1 and 2 molecules, in which all orders from 12th and onward improve upon CCD by a margin of over 5 mH. The improvement is less notable among molecules with row 3 atoms, where the same comparison only yields a single mH improvement. Together, this results in an overall improvement of 3.3 mH in the energies of the double methods, despite the lack of the disconnected-cluster contributions allowing for indirect excitations related to  in the CCD amplitudes.

Moving to the comparison of CCSD, it is useful to use the oo:DCM(*N*) values not just for their quantitative improvement but also due in part to their approximate Brueckner-like nature that ameliorates orbital relation effects, a property already inherent to CCSD with its orbital insensitivity. For the row 1 and 2 containing molecules, oo:DCM(*N*) of 12th order and higher improves the energies by a margin of over 5 mH, while across the entire set this value is a more modest value of just under 2 mH due to the smaller improvements in the row 3 molecules. Whether the smaller improvements for row 3 containing molecules are a feature of the summation scheme or the moments approach more generally is not clear without additional investigation. It is worth noting that values of oo:DCM(*N*) after the 12th order are superior relative to CCSD for 49 of the 55 molecules in the G1 test set. Most of the rmsd error stems from a few systems in which the errors are high, such as the previously mentioned SO_2_. In fact, the successful cases are significant enough for oo:DCM(*N*) to outcompete even CCSD(T) in 15 of the systems tested here.

We also note that the same trend of rising values found in the order-by-order DCM(*N*) and oo:DCM(*N*) energy analysis for individual cases holds true in a statistical sense as well. There is some variation in the solutions, but DCM(14) exhibits the smallest rmsd in both the HF and the OO cases as energies begin to oscillate. Despite this, anything 12th order and higher is no more than 0.21 mH above this energy error for both sets of reference determinants.

As mentioned above, DCM(*N*) scales as the same polynomial power of the system size as the CCD and CCSD equations. Specifically, the construction of (*ij*∥*ab*)_*n*_ is needed to assemble μ_2*N*–1_^D^, and making (*ij*∥*ab*)_*N*_ involves *N* – 1 particle ladder contractions, which roughly makes DCM(*N*) as costly as *N* – 1 iterations of the CCD amplitudes. While the pilot code has not undergone heavy optimization, it may still be useful to mention preliminary timings in constructing the amplitudes versus the DCM(*N*). For the largest system, Si_2_H_6_, CCSD yields 81.3 s per cycle, for a total of 650.6 s in solving the CC equations. By comparison, DCM(*N*) required 42.6 s per cycle, resulting in DCM(14) being just under the CCSD timing at 596.5 s. We can anticipate that when CCSD convergence is slow, the DCM(*N*) models are a fairly straightforward way to evaluate correlation energies with comparable, or even improved, values due to its single shot nature.

### 4.7 N_2_ Dissociation

The potential energy surface corresponding to breaking the triple bond of N_2_ has served as a benchmark case in the evaluation of new correlation methodologies such as CI and CC studies through very high orders, and new multireference CC approaches, including the many different flavors of Fock-space MRCC, state-universal MRCC, and valence-universal MRCC.^93−95^ N_2_ has several valuable properties for benchmarking, beginning with the need to treat both dynamic and static correlations along the dissociation coordinate. While fairly well described by a single determinant at the equilibrium geometry, the triple bond dissociation requires the interaction of six active electrons to recouple the two ^4^N atoms. N_2_ is also an excellent case for testing spatial and spin symmetry (breaking) as a function of bond-breaking. While low-order CC techniques fail qualitatively when using a spin-restricted reference determinant, the use of a spin-polarized reference allows one to obtain quantitative accuracy at dissociation at the cost of losing the spin purity inherent to the true wave function.

**Figure 7:**
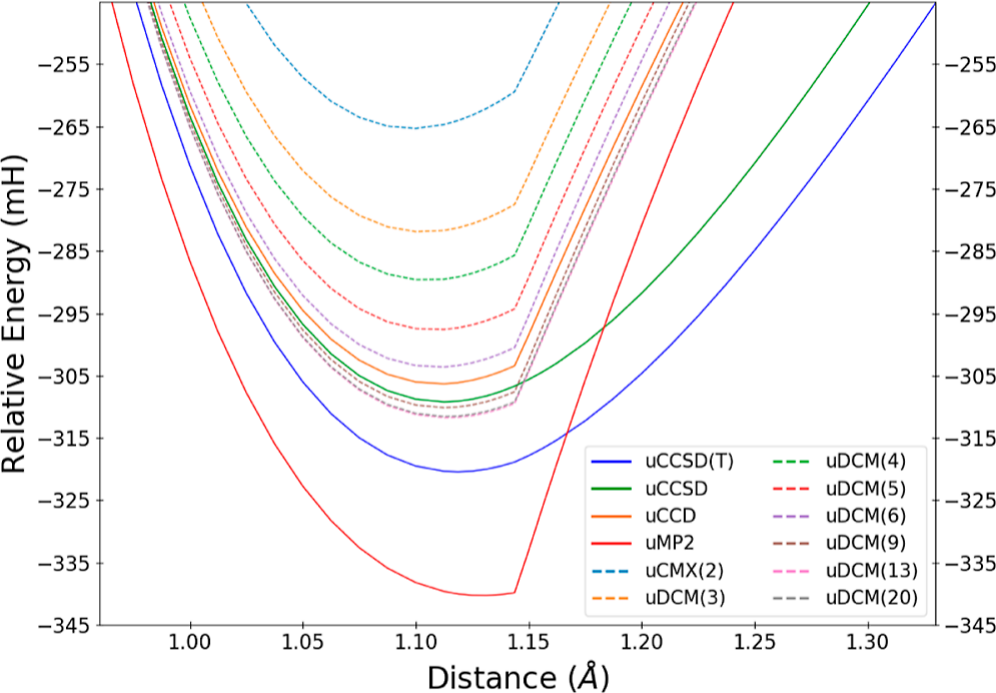
Approximations to the total energy of N_2_ in the vicinity of the equilibrium geometry with a UHF reference for correlation methods, including representatives of the uDCM(*N*) sequence. Energies are reported relative to the two ^4^N atoms for each method.

Figure 7 shows N_2_ dissociation in the cc-pVDZ basis using a spin-polarized reference determinant, comparing uMP2, uCCD, uCCSD, and uCCSD(T) with the DCM(*N*) sequence. To begin, we focus on the convergence of the sequence near the bottom of the well for the Hartree–Fock case before considering the entire curve or the oo:DCM(*N*) case. For readability, we have omitted some orders, but nevertheless one can see the same trends as before: there is variation of the DCM(*N*) energies on the sub-mH energy scale after DCM(11). As usual, the traditional purely double methods of uMP2 and uCCD both exhibit a very visible first derivative discontinuity^96^ at the Coulson–Fischer point, while uCCSD and uCCSD(T) are both able to circumvent this behavior (at least on the graphical scale) via the inclusion of singles. Lacking orbital relaxation due to singles, the uDCM(*N*) family of methods shares the behavior of uMP2 and uCCD with a pronounced first derivative kink at all orders. However, the actual energetic predictions of the uDCM(*N*) sequence follow the trends as seen for the G1 test set: uDCM(*N*) surpasses uCCD by uDCM(6) regardless of whether we are in the unpolarized or spin-polarized regimes.

**Figure 8:**
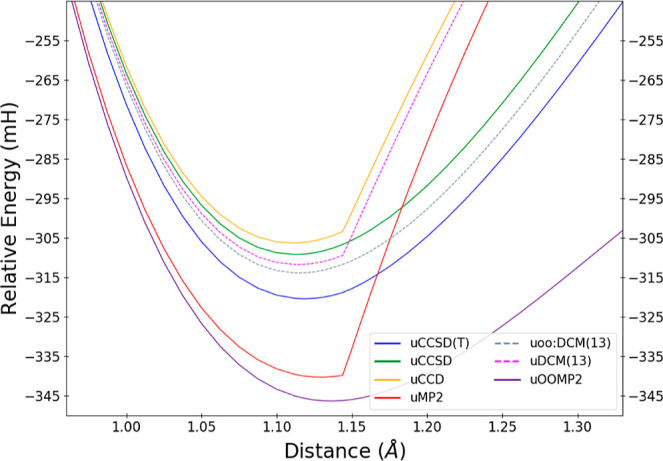
Approximations to the total energy of N_2_ in the vicinity of the equilibrium geometry with a UHF reference for correlation methods, including uDCM(13) and uoo:DCM(13) as high-order representatives of the uDCM(*N*) sequence. Energies are reported relative to two ^4^N atoms for each method.

**Figure 9:**
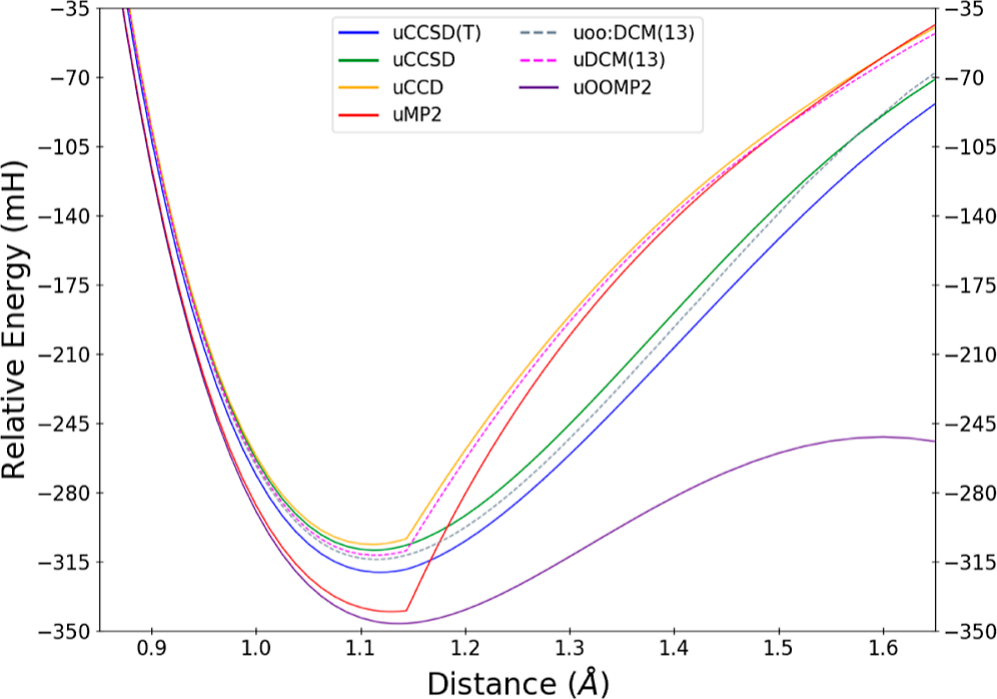
Approximations to the total energy of N_2_ across the potential curve with a UHF reference for correlation methods, including uDCM(13) and uoo:DCM(13) as high-order representatives of the uDCM(*N*) sequence. Energies are reported relative to two ^4^N atoms for each method.

As other work has recently shown,^73^ the inclusion of an orbital-optimized reference delays the onset of the Coulson–Fischer point, and one may recover a better qualitative description within the well. Combined with the performance in energetics, this makes uoo:DCM(*N*) an excellent candidate to evaluate in the same way. As uDCM(13) is the lowest order to reach the convergence limit, we now focus on only this order for both sets of orbitals to make the trend more apparent. Figure 8 shows results similar to above near the well, while Figure 9 shows the behavior up through the qualitative failure and through the point where the uOOMP2 curve turns over (note that uOOMP2 does not exhibit a Coulson–Fischer point as has been extensively discussed elsewhere^73,97^). The uoo:DCM(*N*) curve does, in fact, recover the correct qualitative behavior near equilibrium and well after Hartree–Fock’s spin-polarization transition.

Interestingly, even as the OOMP2 curve begins to turn over, the uoo:DCM(13) results are able to delay this to be more in line with the uCC methods. By the time the former reaches its maxima, this is no longer the case, and both begin to diverge after this point. However, it is notable that the moment’s approach delays the turning point as well. Of course, while the uDCM(13) does have its discontinuity, the curve does not diverge. Additionally, uoo:DCM(13) is able to surpass quantitatively all methods here relative to CCSD(T), now resting at 6.4 mH away rather than CCSD’s 11.2 mH.

## 5 Conclusions

The purpose of this work was to revisit the CMX in order to introduce an approximation analogous to those that have been successful in widely used single-reference correlation methods, such as CC theory. The exact moments (powers of the Hamiltonian) that enter the CMX expressions have compute costs that increase incredibly steeply with the power, which makes these otherwise attractive noniterative methods unfeasible in practice. To circumvent the computational bottleneck, we decided to evaluate the moments using only the double part of the Hilbert space that is orthogonal to a single reference such as the Hartree–Fock determinant or an approximate Brueckner determinant. We call this approach the DCM expansion, and, via the CMX framework, it gives rise to a tractable sequence of DCM energies, DCM(*N*), for *N* = 1...∞. Beyond *N* = 1, each DCM(*N*) energy can be evaluated with computed cost that scales the same as an iteration of the CCD or CCSD equations. The fact that DCM(*N*) energies are constructed from connected moments allows the method to retain the important property of size extensivity of the exact CMX(N) energies, yet more work is needed to determine if more sophisticated ansatzes or non-CMX(*N*) approximate moment methods may allow strict separability.

The DCM(*N*) methods have been implemented to employ single-references that can be either Hartree–Fock or approximate Brueckner from orbital-optimized MP2 (oo) and can be either spin-restricted or unrestricted. The resulting methods were then assessed on the correlation energies of the 55 small molecules (and radicals) in the G1 data set against virtually exact results from selected configuration interaction, as well as standard MP2, CCD, CCSD, and CCSD(T). Interestingly, we observe that DCM(*N*) for *N* > 10 performs quantitatively well relative to infinite order doubles methods such as CCD and CCSD. These are perhaps the first tractable calculations using the moment approach that appear potentially viable for chemical applications. Statistically, the oo:DCM(*N*) energies outperform CCSD for the correlation energy, while the Hartree–Fock-based DCM(*N*) energies outperform CCD for the correlation energy. Examination of both individual and collective results shows generally smooth convergence patterns with respect to limiting values by DCM(11–14).

The DCM(*N*) methodology looks useful already although we stress that it is important to explore questions of numerical stability and basis set extension carefully in future work. If those results are positive, as seems quite likely, then there are practical ways in which the methodology can be systematically improved. First, one may refine the approximation of the moments to include further contributions. In particular, one can easily imagine the construction of SDCM(*N*) by including the singles contributions to μ_2_ and higher. Additionally, there are opportunities to further optimize the implementation. For example, the most costly step in DCM(*N*) is the contraction of the particle ladder terms, and the use of the tensor hypercontraction (THC) formalism would bring the scaling of DCM(*N*) down to  with molecule size as in the case of MP3.^98^ There are likewise opportunities to consider more efficient implementations of higher connected contributions, such as triples. For example, the μ_4_ connected triples may be calculated in *O*(*M*^6^) time unlike its analogue in the MP4 case where a 6-index denominator results in its most expensive contribution scaling as *O*(*M*^7^). Though this specific contribution is still relatively intractable at *O*(*v*^6^) (where *v* is the number of virtual orbitals), the potential factorizability of the method provides further opportunities for the use of THC and resolution-of-the-identity (RI).

Especially in situations that exhibit an interplay of strong and weak correlations, the DCM(*N*) approach may be a blueprint for further refinement of more sophisticated yet still low-cost references, such as strongly orthogonal geminal wave functions^99^ or the coupled cluster valence bond (CCVB).^100,101^ Indeed the prior success seen using low-order CMX with APSG and CAS reference wave functions suggests that there is potential for new progress in this direction, based on retaining the affordability and promising accuracy that is a key feature of DCM(*N*).
